# Temporary Spinal Cord Stimulation for Herpes Zoster With Myelitis: A Case Series

**DOI:** 10.7759/cureus.55979

**Published:** 2024-03-11

**Authors:** Reon Kobayashi, Asae Taketomi, Eiko Hara, Hitoshi Mera, Katsunori Oe

**Affiliations:** 1 Anesthesiology, Showa University School of Medicine, Tokyo, JPN

**Keywords:** zoster-associated pain, spinal cord stimulation, postherpetic neuralgia, myelitis, herpes zoster

## Abstract

Introduction*:* Preventing the development of postherpetic neuralgia (PHN), the most prevalent and severe complication of herpes zoster (HZ), is vital. Recently, it has been suggested that using temporary spinal cord stimulation (tSCS) for 10-14 days can improve HZ-associated pain (ZAP) and prevent PHN. However, myelitis complicates HZ. Permanent SCS has been successful in treating neuropathic pain induced by postoperative transverse myelitis of the spine that has not responded to traditional multidisciplinary treatment. However, it is unknown whether tSCS can reduce ZAP complicated with myelitis.

Methodology*:* Between January 2020 and April 2022, all patients with HZ who visited our pain clinic with spinal cord edema and who underwent tSCS were enrolled in this study; their medical records were retrospectively examined. Pain intensity was assessed at baseline (before initiating interventional procedures), just before tSCS, after tSCS removal, and one and three months after tSCS.

Results*:* Twelve patients were enrolled. The mean Numerical Rating Scale (NRS) was 7.9 ± 1.6 at baseline (before interventional procedures), 6.8 ± 2.2 before tSCS (after interventional procedures), and 3.5 ± 2.4 after tSCS. Compared with before tSCS, the mean NRS decreased to 3.3 ± 2.3 after tSCS (*P *= 0.0004). The mean NRS changes with interventional procedures before and after tSCS were -1.2 ± 2.2 (*P* = 0.0945) and 3.3 ± 2.3 (*P *= 0.0004), respectively; the change after tSCS was significantly higher (between-group difference: -2.1 ± 3.7; *P *= 0.0324).

Conclusions*:* Temporary SCS alleviated pain in cases of shingles with myelitis refractory to interventional therapy. Even in cases with myelitis, tSCS for ZAP remains an effective way to prevent PHN.

## Introduction

Shingles (herpes zoster [HZ]) is caused by the reactivation of latent varicella-zoster virus (VZV) infection, and approximately 30% of people will develop the condition during their lifetime [1]. Based on the time from the initial appearance of the rash, shingles pain is broadly classified as acute HZ pain the first month after the onset of rashes, subacute HZ pain between one and three months after the onset of rashes, and postherpetic neuralgia (PHN) three months or later after the onset of rashes. These stages are collectively referred to as HZ-associated pain (ZAP) [2].

The most common and serious complication of HZ is considered to be PHN [3]. PHN is resistant to several treatments and irreversibly and significantly reduces a patient’s activities of daily living [4]. Therefore, it is important to prevent the transition of HZ to PHN through appropriate pain control. Other complications of HZ include myelitis, cerebral infarction due to vasculitis, meningoencephalitis, sensory disturbance, and facial paralysis (Hunt syndrome) [5,6], of which the incidence of clinical HZ myelitis has been reported to be 0% to 0.8% in both the general population and immunosuppressed patients [7-9].

Symptoms of myelitis are characterized by acute or subacute onset of neurologic signs and symptoms consisting of motor, sensory, and/or autonomic dysfunction. The most common initial symptoms are sensory changes, weakness, and pain in 39%, 25%, and 22% of cases, respectively [10]. HZ myelitis, which does not present with autonomic symptoms such as dyskinesia or vesicorectal disturbances, but is mainly associated with sensory disturbance and pain, is also seen [11].

In general, the most serious sequela of myelitis is the development of chronic neuropathic pain, which severely limits the patient’s independence and adversely affects the quality of life. Because treatment for the pain of myelitis has not been established, and it is considered difficult to treat [12], myelitis associated with shingles may convert to PHN.

Spinal cord stimulation (SCS) is a last-resort treatment for patients with chronic neuropathic pain that relieves pain by stimulating the dorsal column with electricity after inserting a lead into the epidural space [13]. SCS is often implanted permanently in the treatment of PHN when conservative therapy is ineffective and has been reported to be effective in reducing various types of pain, including complex regional pain syndrome (CRPS) and lumbar surgical pain syndrome [14]. There have been reports of permanent SCS being effective for neuropathic pain caused by postoperative transverse myelitis of the spine [15].

Recently, it has been reported that temporary SCS (tSCS) for 10-14 days, without permanent SCS implantation, may rapidly relieve ZAP and prevent PHN in ZAP, which is resistant to conventional multidisciplinary treatment [16-18]. However, these reports do not consider the presence of myelitis. HZ complicated by myelitis, for which there is no established treatment, may well be converted to PHN.

Twelve patients with ZAP and myelitis who were refractory to interventional treatments underwent tSCS, and their records were studied retrospectively.

## Materials and methods

This study was conducted per the ethical principles of the Declaration of Helsinki and ethical guidelines for life science and medical research involving human subjects and was approved by the Showa University Ethics Committee (approval number: 22-018-B) on December 22, 2022. Twelve patients who consulted our pain clinic between January 2020 and April 2022 with spinal cord edema and who had undergone tSCS were enrolled in this study, and their medical records were retrospectively examined. The method of obtaining consent for the use of medical record information was opt-out via a website.

The primary endpoint was the intensity of pain at the site of the skin rash. Pain severity was assessed using an 11-point numerical rating scale (NRS) ranging from 0 (no pain) to 10 (severe pain) at baseline (before initiating interventional procedures), immediately before tSCS, after tSCS removal, and one and three months after tSCS. The secondary endpoints included patient characteristics (sex, age, body mass index [BMI], the location of the rash, sensory disturbance on the rash, the presence of allodynia, availability of antivirus therapy, corticosteroid use, and type of interventional procedures before tSCS), time from onset of HZ to treatment and from the start of treatment to tSCS, tSCS characteristics (type of tSCS stimulation, location of the top of the tSCS lead, and complications), date of acquiring magnetic resonance imaging (MRI) of myelitis, and changes in pharmacotherapy. Interventional procedures include transforaminal epidural steroid injection and/or epidural block (injection) and/or stellate ganglion block and/or intercostal nerve block, and pulsed radiofrequency to the responsible nerve root.

tSCS was implanted by three physicians: two were pain clinic specialists and one was a trainee. All patients were positioned in the prone position, and the puncture point was marked under fluoroscopy before the procedure. The target site of tSCS treatment was determined with reference to the skin segment of the HZ; at least 50% of the painful area was covered by a pleasant sensation. Two 1 × 8 or 1 × 16 electrode stimulation leads (Vectris SureScan MRI 1 × 8 compact lead, Model 977A2, Medtronic, Minneapolis, MN; or Octrode lead, Model 3186, Abbott, Plano, TX; or 1 × 16 electrode stimulation Infinion CX lead, Boston Scientific, Marlborough, MA) were implanted in the epidural space under fluoroscopic guidance with local anesthesia. The first lead was positioned at the spinous process margin (right or left), and the second lead was positioned on the affected side of the first to ensure that the target area was covered. After lead insertion, the patients received tSCS for 14 days.

Data normality was evaluated using the Shapiro-Wilk test and presented as mean ± standard error for continuous variables. NRS scores were compared using a corresponding t-test when normally distributed and the Wilcoxon signed-rank test when not normally distributed. All statistical analysis was performed using JMP Pro version 16.2.0 (SAS Institute, Cary, NC). *P-*values < 0.05 were considered statistically significant.

## Results

During the observation period, 236 patients had HZ, among whom 29 patients underwent MRI, and all 15 patients with spinal cord edema received tSCS. One patient was unable to assess pain, one patient had an insufficient observation period, one patient’s pain improved, and 12 patients were enrolled in the study. Table 1 summarizes the basic characteristics of the enrolled patients. Eight and four patients had shingles on the chest and neck, respectively; the myelitis MRI scans were unilateral in 11 patients and transverse in one patient. Ten patients reported hypoesthesia of the affected cutaneous segments; all patients reported allodynia. All patients received antiviral therapy; five patients received steroids. Interventional procedures were performed in all cases, and 10 patients underwent nerve root pulsed radiofrequency at least once before tSCS.

**Table 1:** Patient characteristics. ^*^Sensory disturbance with normal as 10. ^A^Transforaminal epidural steroid injection or/and epidural block (injection) or/and stellate ganglion block or/and intercostal nerve block. ^B^Pulsed radiofrequency to the responsible nerve root. F, female; M, male; C, cervical spinal nerve; T, thoracic spinal nerve; r, right; l, left; tSCS, temporary spinal cord stimulation

No.	Age (year)	Sex	BMI (kg/m^2^)	Location of rash	Right or left	Sensory disturbance on the rash^＊^	Baseline pain score (before interventional procedures)	Allodynia	Antiviral therapy	Corticosteroid	Interventional procedures before tSCS
1	86	F	21.8	T9	r	4	8	+	+	-	^A^
2	79	M	22.0	T10	r	4	7	+	+	-	^A, B^
3	79	M	23.7	C2	r	3	7	+	+	-	^A, B^
4	75	F	24.4	C4	l	1	9	+	+	+	^A^
5	70	M	22.8	T4	l	10	7	+	+	+	^A, B^
6	73	M	21.5	T4	r	8	8	+	+	+	^A, B^
7	72	F	23.7	C8	r	5	10	+	+	-	^A, B^
8	78	F	26.7	C3	l	8	10	+	+	+	^A, B^
9	53	M	26.6	T4	l	7	5	+	+	+	^A, B^
10	58	F	11.1	T9	r	0	10	+	+	-	^A, B^
11	82	M	22.1	T5	l	12	7	+	+	-	^A, B^
12	77	M	19.6	T5	r	8	7	+	+	-	^A, B^
Mean	74 ± 11	M 45%; F 55%	22.0 ± 4.2			5.8 ± 3.6	7.9 ± 1.9				

Table 2 shows the time from HZ onset to interventional procedures and tSCS. Interventional procedures were performed for ZAP in all patients before the initiation of tSCS. The time from HZ onset to tSCS was 72.9 ± 25.9 days, and the time from initiation of interventional procedures to tSCS was 44.8 ± 26.6 days.

**Table 2:** Time from the onset of HZ to treatment and from the start of treatment to tSCS in days. HZ, herpes zoster; tSCS, temporary spinal cord stimulation

No.	Disease duration before interventional procedures (day)	Disease duration before tSCS (day)	Duration between interventional procedures and tSCS (day)
1	30	51	21
2	28	53	25
3	37	64	27
4	26	42	16
5	34	133	99
6	35	75	40
7	24	54	30
8	26	58	32
9	37	73	36
10	19	82	63
11	41	105	64
12	1	85	84
Mean	28.2 ± 10.7	72.9 ± 25.9	44.8 ± 26.6

Table 3 summarizes the tSCS characteristics. Two patients underwent tonic stimulation, and 10 patients underwent BurstDR stimulation. No serious adverse events were observed. The most common complication during tSCS was lead migration. In three cases, the lead moved more than one vertebra. The leads were shifted to two vertebrae lower in case 5 and one vertebra lower in cases 9 and 10. However, stimulation adjustments were repeated frequently to match the pain, and stimulation was performed in the appropriate position to the maximum extent throughout the period.

**Table 3:** tSCS characteristics. tSCS, temporary spinal cord stimulation; C, cervical spinal nerve; T, thoracic spinal nerve

No.	tSCS stimulation	Location of top of the tSCS lead	Complication
1	Tonic	T3	None
2	Tonic	T3	None
3	BurstDR	C2	None
4	BurstDR	C2	None
5	BurstDR	C7	Lead migration
6	BurstDR	C7	None
7	BurstDR	C2	None
8	BurstDR	C1	None
9	BurstDR	T1	Lead migration
10	BurstDR	T6	Lead migration
11	BurstDR	T1	None
12	BurstDR	C7	None

**Figure 1:**
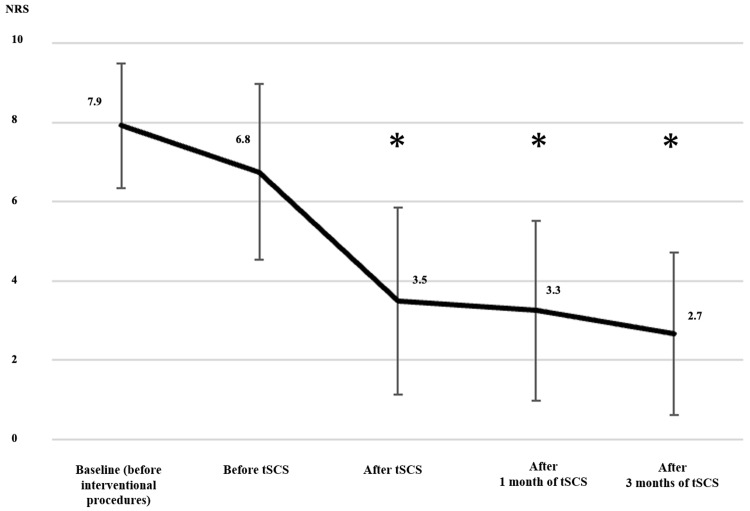
Change in NRS from baseline. ^*^Significantly lower than NRS before tSCS (*P *< 0.05). NRS, Numerical Rating Scale; tSCS, temporary spinal cord stimulation

The changes in NRS are shown in Table 4. The mean NRS was 7.9 ± 1.6 at baseline (before interventional procedures), 6.8 ± 2.2 before tSCS (after interventional procedures), and 3.5 ± 2.4 after tSCS. Compared with before tSCS, the mean NRS decreased to 3.3 ± 2.3 after tSCS (*P *= 0.0004). The mean NRS changes between interventional procedures and before and after tSCS were -1.2 ± 2.2 (*P *= 0.095) and -3.3 ± 2.3 (*P *= 0.0004), respectively; the tSCS change was significantly higher (between-group difference: -2.1 ± 3.7, *P *= 0.0324). Compared with before tSCS, a significant decrease was observed after tSCS and one and three months after tSCS (*P *< 0.05) (Figure 1). Case 10 was unable to work because of pain; therefore, tSCS was performed. However, NRS was denoted as 3.

**Table 4:** Changes in NRS. MRI, magnetic resonance imaging; tSCS, temporary spinal cord stimulation; NRS, numerical rating scale; m, month

No.	Baseline (before interventional procedures)	Before tSCS	After tSCS	After 1 m of tSCS	After 3 m of tSCS	Final follow-up
1	8	8	5	7	5	6 (12 m after tSCS)
2	7	8	2	5	4	3 (16 m after tSCS)
3	7	9	6	3	3	3 (10 m after tSCS)
4	9	9	8	5	3	4 (9 m after tSCS)
5	7	6	4	4	4	4 (12 m after tSCS)
6	8	8	6	5	6	3 (10 m after tSCS)
7	10	5	2	0	0	0 (6 m after tSCS )
8	10	10	1	1	0	0 (6 m after tSCS )
9	5	3	1	0	0	0 (3 m after tSCS )
10	10	5	4	5	4	3 (12 m after tSCS)
11	7	4	1	2	1	1 (3 m after tSCS )
12	7	6	2	2	2	2 (3 m after tSCS )
Mean	7.9 ± 1.6	6.8 ± 2.3	3.5 ± 2.4	3.3 ± 2.3	2.7 ± 2.1	

**Figure 2:**
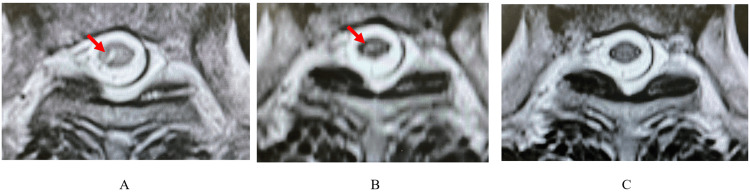
Change in myelitis of case 2. Arrows indicate unilateral edema in case 2. (A) Before tSCS: one month after HZ onset; (B) thinning: 19 months after HZ onset; (C) disappeared: 30 months after HZ onset. The high-signal region within the spinal cord is gradually thinning. tSCS, temporary spinal cord stimulation; HZ, herpes zoster

**Figure 3:**
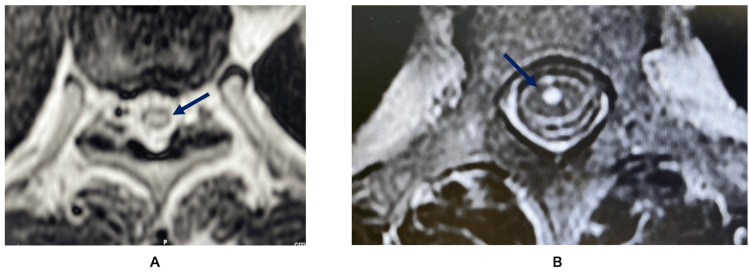
MRI image of edema. (A) Arrows indicate a bilateral edema in case 1; (B) arrows indicate an edema of the anterior horn of the spinal cord in case 9. MRI, magnetic resonance imaging

The MRI findings of spinal edema are shown in Table 5, with representative images in Figure 2. Spinal cord edema was often observed throughout the unilateral gray matter of the spinal cord. However, bilateral edema was observed in case 1 (Figure 3A). The edema was closer to the anterior horn rather than the dorsal horn (Figure 3B).

**Table 5:** MRI data of myelitis. m, month; C, cervical spinal nerve; T, thoracic spinal nerve; MRI, magnetic resonance imaging

No.	Crossing myelitis or unilateral	Myelitis level	High-intensity area/spinal cord (%)	Change in the high-intensity area (%)	Myelitis outcomes
1	Crossing	T4-T10	24.7	None	None
2	Unilateral	T7-T10	13.9	13.5% and thinning after 19 m of shingles onset	Disappeared 30 m after onset of shingles
3	Unilateral	C1	10.7	3.7% and thinning after 6 m of shingles onset	Disappeared 12 m after onset of shingles
4	Unilateral	C3	8.0	None	None
5	Unilateral	T1-T2	12.2	Disappeared 16 m after onset of shingles	None
6	Unilateral	T3-T4	13.7	None	None
7	Unilateral	T6	3.9	0.9% and thinning after 6 m of shingles onset	None
8	Unilateral	C2	7.3	7.1% and thinning after 1 m of shingles onset	None
9	Unilateral	T2	11.7	None	None
10	Unilateral	T5-T7	9.0	None	None
11	Unilateral	T3-T5	10.6	None	None
12	Unilateral	T3	19.2	None	None

Additional MRI was performed after tSCS in five cases (cases 2, 3, 5, 7, and 8). In case 2, the proportion of high-signal areas indicating edema to the entire spinal cord did not change much from HZ onset to 19 months (13.9%-13.5%); however, the intensity of the high signal decreased. NRS was 7 at onset and 2 and 4 at 19 and 30 months, respectively. In case 3, the proportion of high-signal areas decreased from 10.3% to 3.7% from HZ onset to six months, and the signal intensity decreased. NRS was 8 at onset and 3 at both six months and one year. In case 5, the proportion of high-signal areas was 12.2% at HZ onset and had disappeared completely by 16 months; NRS was 7 and 4 at HZ onset and 16 months, respectively. In case 7, the proportion of high-signal areas decreased from 3.9% to 0.9% from HZ onset to six months, and the signal intensity was reduced. NRS was 8 and 0 at onset and six months, respectively. In case 8, the proportion of high-signal areas decreased from 7.3% to 7.1% from HZ onset to one month, and the signal intensity was reduced. NRS was 10 and 0 at onset and one month, respectively.

Table 6 shows the changes in pharmacotherapy. The medications used included opioids in zero cases, tramadol in five cases, pregabalin in nine cases, mirogabalin in three cases, duloxetine in seven cases, non-steroidal anti-inflammatory drugs in two cases, and acetaminophen in six cases before tSCS. The medications were changed after tSCS according to pain; the dosages of neuropathic pain medications were reduced in more cases at three months after tSCS, while the doses of loxoprofen and tramadol were increased in cases 1 and 2, respectively.

**Table 6:** Pharmacotherapy. Values with backslashes ( / ) represent three months before/after temporary spinal cord stimulation (tSCS) removal. NSAID, non-steroidal anti-inflammatory drug

No.	Tramadol (mg/day)	Pregabalin (mg/day)	Mirogabalin (mg/day)	Duloxetine (mg/day)	NSAIDs (mg/day)	Acetaminophen (mg/day)
1	None	0/25	10/0	20/20	0/Loxoprofen 60	None
2	0/100	150/150	None	20/20	None	None
3	50/50	None	15/10	40/0	Lornoxicam 4/0	None
4	None	150/0	0/10	20/20	None	500/0
5	75/75	175/150	None	None	None	None
6	75/75	150/150	None	20/20	None	1250/1250
7	None	150/50	None	20/0	Loxoprofen 180/0	None
8	None	225/0	None	None	None	1200/0
9	None	250/50	None	None	None	None
10	None	50/25	None	None	None	1600/1600
11	50/0	125/100	None	40/20	None	2000/0
12	150/25	None	20/20	None	None	3300/0

## Discussion

Temporary SCS improved pain scores even in cases of shingles with myelitis refractory to interventional therapy. tSCS improved ZAP in 12 patients with myelitis, where the NRS was significantly lower after tSCS than before tSCS, and the change after tSCS was significantly greater than that after interventional procedures. The analgesic mechanism of tSCS is more closely linked to the central nervous system than that of interventional techniques, suggesting that even pain of spinal origin may be relieved by tSCS.

Dong et al. performed tSCS on 46 patients who presented with acute and subacute herpetic pain and who had failed conventional therapy [17]. Their study showed that ZAP, along with analgesics, significantly decreased compared to pre-tSCS, despite the failure of conventional therapy. Moriyama performed tSCS for ZAP in the thoracic region in 14 patients who showed no improvement in pain with a combination of continuous epidural block and pharmacotherapy [19]. The author reported that the change in NRS was greater for tSCS than for interventional procedures, indicating that tSCS improves pain in response to ZAP resistance to interventional therapy. In an observational study by Huang et al., tSCS was also performed in 99 patients with ZAP who had failed pharmacological and interventional therapy [16]. They reported that performing tSCS in ZAP within 80 days of onset prevented postherpetic neuralgia. However, the VAS was not reduced to less than 2 in the PHN group who underwent tSCS more than 90 days after onset.

Although their participants were not asked about the presence or absence of findings of spinal edema, the results of their study support our finding that tSCS provided analgesia to patients who did not experience significant pain relief with interventional procedures and may do so even in patients with ZAP with myelitis.

As there have been no previous reports on tSCS for ZAP with myelitis, so comparisons with other cases are not possible. On the other hand, there have been reports of myelitis improving with pharmacotherapy and interventional procedures. Giménez-Milà et al. reported the case of a 73-year-old woman diagnosed with CRPS of the upper extremity in addition to PHN with myelitis [12]. They performed two sympathetic nerve blocks, and NRS improved from 8 to 2. Farhat et al. presented five cases of myelopathy successfully treated with antiviral drugs [6]. All patients in the present study had already received antivirals, and in six cases, steroids were also administered, which may have prevented myelopathy from developing. The only symptoms were pain and sensory insensitivity.

In general, pain caused by myelitis is often resistant to current drug therapies such as opioids, antidepressants, and anticonvulsants, and there is insufficient clinical evidence that SCS is effective in reducing pain [12]. However, possible reasons for the lower pain in this case are, first, that the myelitis was reversible and the physiological spinal dorsal cord was preserved, and second, that BurstDR stimulation was used in many cases.

Tasker et al. measured the response to SCS in a study of 127 central pain patients with spinal cord lesions and concluded that the analgesic effect of SCS is diminished when severe spinal dorsal cord atrophy occurs [20]. In the present case, the edema was only unilateral and the changes were reversible, as shown in the MRI data in Table 5, suggesting that the physiological spinal dorsal cord was preserved. Therefore, tSCS was considered effective.

However, if the posterior column is highly inflamed, the conclusion that the spinal cord injury (SCI) was incomplete may not fully justify the improvement in pain. In this series in particular, there were many cases at the upper cervical (nos. 3 and 8) and thoracic (nos. 1, 2, 5, 6, 9, 10, 11, and 12) spine levels where muscle weakness was difficult to assess, making it difficult to evaluate the severity of the disease.

Therefore, another reason why tSCS reduced pain even in the presence of myelitis may be that BurstDR stimulation was more commonly used. Differences in the analgesic mechanisms between BurstDR stimulation and conventional tonic stimulation have been noted; the reported analgesic mechanisms of tonic stimulation include activation of the descending pain inhibitory system by gate control theory and induction of inhibitory postsynaptic potentials in dorsal horn neurons [21]. However, these require undamaged afferent pathways to activate the dorsal column and transmit pain signals from the periphery to the central nervous system.

However, it has been reported that the analgesic mechanism of BurstDR stimulation has an analgesic mechanism that does not involve the dorsal column; that is, direct stimulation of C-fiber-derived wide dynamic range (WDR) neurons in the dorsal horn of the spinal cord [22] inhibits WDR neurons without mediating the γ-aminobutyric acid system within the dorsal column [23]. Furthermore, the anti-inflammatory effects of BurstDR stimulation include an increase in the peripheral anti-inflammatory cytokine interleukin-10 (IL-10) [24] and an increase in neuropeptides due to changes in immunity [25]. In this study, BurstDR stimulation was used in 10 of 12 cases, which may have improved pain even in more disabling myelitis, such as with atrophy of the dorsal column.

On MRI, the high signal indicating myelitis gradually decreased. This is considered the healing process of HZ. In case 2, the pain improved even though the proportion of high-signal areas remained almost the same. Therefore, high-signal areas did not seem to be closely correlated with pain intensity. However, a reduction in the signal intensity and pain was observed simultaneously in all cases. Therefore, it is impossible to say that there is no connection between pain and signal intensity. At present, evaluating the gray matter of the spinal cord is a manual process and is considered difficult due to rater bias [26]. In the future, rigorously assessing the signal intensity and area of myelitis may provide more insight into the mechanism by which SCS reduces pain in myelitis.

This study has a few limitations. This case series only included 12 cases. First, the inherent weaknesses of the retrospective data collection are noted. There was no assessment of important aspects of pain other than NRS. There were differences in time to tSCS implantation, and MRI was not performed in all cases after tSCS. Second, pulsed radiofrequency was performed before tSCS. Therefore, the decrease in NRS during tSCS treatment may be due to the combined effects of these treatments. Third, ZAP is a type of pain that may resolve spontaneously. However, the improvement of pain in this case was considered to be due to tSCS because a rapid decrease in pain was observed within two weeks of the procedure. Fourth, we did not blindly control patients in the group to see if their pain worsened by turning off the stimulus during the trial. This is because the presence and duration of carryover effects of SCS have not been fully established. Fifth, since there was no control group, it is unclear whether tSCS for ZAP with myelitis has the same analgesic effect as tSCS for ZAP without myelitis. Therefore, further randomized controlled trials with more patients are needed.

## Conclusions

Temporary SCS improved pain scores in shingles with myelitis refractory to interventional therapy. Even in cases with myelitis, tSCS for ZAP remains an effective ways to prevent PHN.

## Appendices

Abbreviations

body mass index (BMI)

complex regional pain syndrome (CRPS)

herpes zoster (HZ)

herpes zoster-associated pain (ZAP)

magnetic resonance imaging (MRI)

numerical rating scale (NRS)

postherpetic neuralgia (PHN)

spinal cord injury (SCI)

spinal cord stimulation (SCS)

temporary spinal cord stimulation (tSCS)

varicella-zoster virus (VZV)

wide dynamic range (WDR)
